# Supplementary material for: Psychological therapies for adolescents with borderline personality disorder (BPD) or BPD features—A systematic review of randomized clinical trials with meta-analysis and Trial Sequential Analysis
Source: PLoS One. 2021 Jan 14;16(1):e0245331. doi: 10.1371/journal.pone.0245331 (PMC7808579; doi:10.1371/journal.pone.0245331)
Supplement: S1 File — (DOCX) [file pone.0245331.s002.docx]

# **S1 Search strategy for Medline**

Medline Ovid

1 Borderline Personality Disorder/

2 ((borderline or border‐line) adj3 (state* or personalit*)).kf,tw.

3 ("Axis II" or "Cluster B" or flamboyant or "F60.3" or "F60.30" or "F60.31").kf,tw.

4 (idealization adj5 devaluation).kf,tw.

5 ((vulnerable or hyperbolic) adj3 temperament).kf,tw.

6 (((unstab* or instab* or poor or disturb* or fail* or weak or dysregulat*) adj3 (self* or impuls* or interperson* or identit* or relationship* or emotion* or affect*)) and (personality or character or PD)).kf,tw.

7 (impulsiv* adj5 (behavio?r or character or personalit*)).kf,tw.

8 (self adj3 (injur* or damag* or destruct* or harm* or hurt* or mutilat*)).kf,tw.

9 (suicidal adj3 behavio?r).kf,tw.

10 (feel* adj3 (empt* or bored*)).kf,tw.

11 (anger adj5 control*).kf,tw.

12 (risk‐taking adj3 behavio?r).kf,tw.

13 or/1‐12

14 randomised controlled trial.pt.

15 controlled clinical trial.pt.

16 randomi#ed.ab.

17 placebo.ab.

18 randomly.ab.

19 trial.ab.

20 groups.ab.

21 drug therapy.fs.

22 or/14‐21

23 exp Animals/ not Humans/

24 22 not 23

25 13 and 24
