# Supplementary material for: Psychological therapies for adolescents with borderline personality disorder (BPD) or BPD features—A systematic review of randomized clinical trials with meta-analysis and Trial Sequential Analysis
Source: PLoS One. 2021 Jan 14;16(1):e0245331. doi: 10.1371/journal.pone.0245331 (PMC7808579; doi:10.1371/journal.pone.0245331)
Supplement: Attachment — Submitted filename: Response to the reviewer.docx [file pone.0245331.s003.docx]

**On revisions to the manuscript**

**Comment #1**

Please ensure that your manuscript meets PLOS ONE’s style requirements, including those for file naming.

**Response**

Thank you for pointing this out. We hope the manuscript and files now meet PLOS ONE’s style requirements

**Comment #2**

Please provide the full electronic search strategy for at least one database, including any limits used, such that it could be repeated.

**Response**

This information has now been added as supplementary information, file S1 Search strategy for Medline

**Comments #3 and #4**

Please ensure that you refer to Figures 2 and 3 in your text as, if accepted, production will need this reference to link the reader to the figure.

We note you have included a table to which you do not refer in the text of your manuscript. Please ensure that you refer to Tables 4 and 5 in your text; if accepted, production will need this reference to link the reader to each Table.

**Response**

Thank you for pointing this out. The figures and tables are now referred to in the text

**Comment**

Please include captions for your Supporting Information files at the end of your manuscript, and update any in-text citations to match accordingly.

**Response**

Thank you, this has now been added.
